# Supplementary material for: Evidence for a hydrogen sulfide-sensing E3 ligase in yeast
Source: Genetics. 2024 Oct 8;228(3):iyae154. doi: 10.1093/genetics/iyae154 (PMC11538405; doi:10.1093/genetics/iyae154)
Supplement: iyae154_Supplementary_Data [file iyae154_supplementary_data.docx]

**Supplementary Materials**

Zane Johnson^1,2^, Yun Wang^1^, Benjamin M. Sutter^1^, Benjamin P. Tu^1*­^

^1^Department of Biochemistry, University of Texas Southwestern Medical Center, Dallas, TX 75390-9038, USA

^2^Present address: Department of Molecular Biophysics & Biochemistry, Yale University, New Haven, CT 06520, USA

*Corresponding Author: [benjamin.tu@utsouthwestern.edu](mailto:benjamin.tu@utsouthwestern.edu)

Supplementary Figures S1-3

Supplementary Tables S1-2

**Figure S1.** Characterization of the faster-migrating proteoform of Met30.

(A) Western blot of yeast treated with 200 μg/ml cycloheximide during sulfur starvation demonstrates that production of the faster-migrating proteoform is dependent on new translation. (B) The faster-migrating proteoform persists after rescue from sulfur starvation when treated with a proteasome inhibitor. Cells were starved of sulfur for 3 h to accumulate the faster-migrating proteoform, and then sulfur metabolites were added back concomitantly with MG132 (50 μM).

(C) The faster-migrating proteoform of Met30 is dependent on Met4. The *met4∆* yeast strain does not produce the second proteoform of Met30 when starved of sulfur.

(D) Western blot analysis of strains expressing either wild type Met30, Met30 Δ1-20aa, or Met30 M30/35/36A. Yeast cells harboring the N-terminal deletion of the first twenty amino acids of Met30 (which contain the first three methionine residues) or have the subsequent three methionine residues (M30/35/36) mutated to alanine do not create faster-migrating proteoforms.

(E) Met30(Δ1-20aa) and Met30(M30/35/36A) strains do not exhibit any growth phenotypes in −sulfur glucose media with or without supplemented methionine. There are also no defects in growth rate following repletion of methionine. Data represent mean and SD of biological triplicates.

**Figure S2.** Identification of key cysteine residues in Met30 involved specifically in sulfur amino acid sensing.

(A) Western blot analysis of Met4 ubiquitination in WT and various Met30 cysteine point mutants in rich and −sulfur media. Lane 3 depicts Met4 re-ubiquitination in response to treatment of sulfur-starved yeast cells with 5 mM DTT for 15 min.

(B) Western blot analysis of Met30 and Met4 ubiquitination status in WT and two cysteine to serine mutants, C414S and C614/616/622/630S, following treatment with 500 µM CdCl_2_.


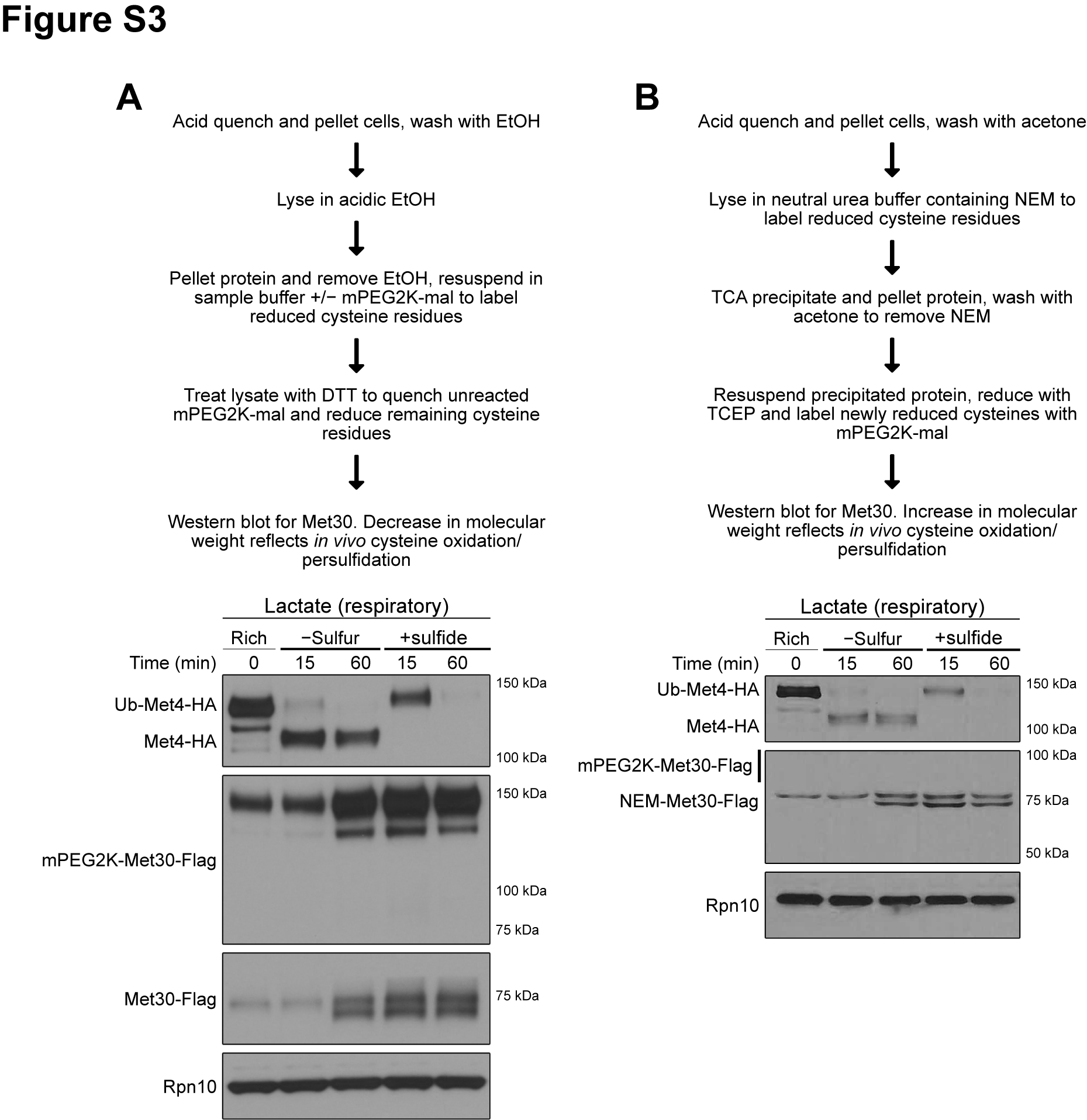


**Figure S3.** Cysteine labeling methods to detect Met30 cysteine oxidation or persulfidation.

(A) Western blot of Met30 over the time course used in Figure 3. Cells were rapidly quenched in acid and lysed in acidic ethanol to preserve the redox state of Met30 cysteine residues. Precipitated protein was resuspended in sample buffer with or without mPEG2K-mal to label reduced cysteine residues in Met30 before reduction with DTT. Unmodified sample was used to blot for Met30 and Rpn10 as controls.

(B) Western blot of Met30 was performed using a modified tag-switch method to detect changes in the redox or persulfidated status of Met30 cysteine residues over the sulfur starvation time course used in Figure 3 by mass shift. Cells were lysed in the presence of the small alkylating reagent NEM to modify all reduced or persulfidated protein thiols in the lysate, followed by acid precipitation of proteins, removal of residual NEM, resuspension and reduction by TCEP, followed by a final alkylation step with mPEG2K-mal. The mPEG2K-mal reagent is expected to add approximately 2 kDa in mass for every oxidized (RSSR, RSOH, etc) or persulfidated (RSSH) cysteine residue.

**Table S1.** Strains used in this study.

| Background | Genotype | Source |
| --- | --- | --- |
| CEN.PK | MATa | ^44^ |
| CEN.PK | MATα | ^44^ |
| CEN.PK | MATa; MET30-FLAG::KanMX | This study |
| CEN.PK | MATa; MET30-FLAG::KanMX MET4-HA::Hyg | This study |
| CEN.PK | MATa; MET30-FLAG::KanMX MET4-HA::Hyg met6Δ::Nat | This study |
| CEN.PK | MATa; MET30-FLAG::KanMX MET4-HA::Hyg str3Δ::Nat | This study |
| CEN.PK | MATa; met30::MET30-C414S-FLAG::KanMX MET4-HA::Hyg | This study |
| CEN.PK | MATa; met30::MET30-C614/616/622/630S-FLAG::KanMX MET4-HA::Hyg | This study |
| CEN.PK | MATa; met30Δ::Phleo HO::MET30-FLAG::Nat MET4-HA::Hyg | This study |
| CEN.PK | MATa; met30Δ::Phleo HO::MET30Δaa1-20-FLAG::Nat Met4-HA::Hyg | This study |
| CEN.PK | MATa; met30Δ::Phleo HO::MET30-M30/35/36A-FLAG::Nat Met4-HA::Hyg | This study |
| CEN.PK | MATa; MET30-FLAG::KanMX MET4-HA::Hyg pdr5Δ::Nat | This study |
| CEN.PK | MATa; met4Δ::KanMX MET30-FLAG::Hyg | This study |
| CEN.PK | MATa; met30::MET30-C201S-FLAG::KanMX MET4-HA::Hyg | This study |
| CEN.PK | MATa; met30::MET30-C374S-FLAG::KanMX MET4-HA::Hyg | This study |
| CEN.PK | MATa; met30::MET30-C426S-FLAG::KanMX MET4-HA::Hyg | This study |
| CEN.PK | MATa; met30::MET30-C436S-FLAG::KanMX MET4-HA::Hyg | This study |
| CEN.PK | MATa; met30::MET30-C439S-FLAG::KanMX MET4-HA::Hyg | This study |
| CEN.PK | MATa; met30::MET30-C455S-FLAG::KanMX MET4-HA::Hyg | This study |
| CEN.PK | MATa; met30::MET30-C528S-FLAG::KanMX MET4-HA::Hyg | This study |
| CEN.PK | MATa; met30::MET30-C544S-FLAG::KanMX MET4-HA::Hyg | This study |
| CEN.PK | MATa; met30::MET30-C584S-FLAG::KanMX MET4-HA::Hyg | This study |
| CEN.PK | MATa; met30::MET30-C614S-FLAG::KanMX MET4-HA::Hyg | This study |
| CEN.PK | MATa; met30::MET30-C616S-FLAG::KanMX MET4-HA::Hyg | This study |
| CEN.PK | MATa; met30::MET30-C584/622S-FLAG::KanMX MET4-HA::Hyg | This study |
| CEN.PK | MATa; met30::MET30-C630S-FLAG::KanMX MET4-HA::Hyg | This study |
| CEN.PK | MATa; MET30-FLAG::KanMX MET4-HA::Hyg met17Δ::Nat met10Δ::Phleo | This study |
| S288C | MATa; MET30-FLAG::KanMX MET4-HA::Hyg | This study |
| S288C | MATa; MET30-FLAG::KanMX MET4-HA::Hyg cys3Δ::Nat | This study |
| S288C | MATa; MET30-FLAG::KanMX MET4-HA::Hyg cys4Δ::Nat | This study |

**Table S2.** Recipe for sulfur-free media.

| salts (g L^-1^) | |
| --- | --- |
| CaCl_2_•2H_2_O | 0.1 |
| NaCl | 0.1 |
| MgCl_2_•6H_2_O | 0.412 |
| NH_4_Cl | 4.05 |
| KH_2_PO_4_ | 1 |
| metals (mg L^-1^) | |
| boric acid | 0.5 |
| CuCl_2_•2H_2_O | 0.0273 |
| KI | 0.1 |
| FeCl_3_•6H_2_O | 0.2 |
| MnCl_2_•4H_2_O | 0.4684 |
| Na_2_MoO_4_•2H_2_O | 0.2 |
| ZnCl_2_•H_2_O | 0.1895 |
| vitamins (mg L^-1^) | |
| biotin | 0.002 |
| calcium pantothenate | 0.4 |
| folic acid | 0.002 |
| inositol | 2 |
| niacin | 0.4 |
| 4-aminobenzoic acid | 0.2 |
| pyridoxine HCl | 0.4 |
| riboflavin | 0.2 |
| thiamine-HCl | 0.4 |

Recipes are derived from^47^.
